# Heart Failure Predictors in a Group of Patients with Myocardial Infarction

**DOI:** 10.3889/oamjms.2016.101

**Published:** 2016-09-06

**Authors:** Sokol Myftiu, Petrit Bara, Ilir Sharka, Artan Shkoza, Xhina Belshi, Edlira Rruci, Gentian Vyshka

**Affiliations:** 1 *Service of Cardiology, University Hospital Centre “Mother Theresa”, Tirana, Albania*; 2 *Biomedical and Experimental Department, Faculty of Medicine, University of Medicine, Tirana, Albania*

**Keywords:** Heart Failure (HF), Acute Myocardial Infarction (AMI), ejection fraction (EF), epidemiology, prognosis

## Abstract

**AIM:**

The present study considers of the prevalence of heart failure (HF) in patients suffering from acute myocardial infarction (AMI) in the University Hospital Centre of Tirana (UHCT) “Mother Theresa”; the demographic and clinical characteristics of the sample during hospitalization; and the main predictors of heart failure occurrence inside the group of patients suffering an AMI.

**MATERIAL AND METHODS:**

During a period of study from 2013-2015 we studied demographic and clinical data from 587 consecutive patients presenting with AMI; Framingham criteria were adopted for classifying patients with HF upon admission.

**RESULTS:**

A Killip class ≥ 2 was the main diagnostic criterion of HF during hospitalisation. HF was identified in 156 patients (26.6%). The subgroup with HF had significant differences when compared with the other patients with regard to age, sex (male), heart rate upon admission, systolic blood pressure on admission, previous episodes of AMI, glycemia on admission, previous antihypertensive treatment, previous revascularization procedures, peripheral vascular disease, chronic renal disease, ejection fraction (EF), anemia, and atrial fibrillation presence. Independent predictors for HF occurrence in the logistic regression model were EF, previous revascularization, peripheral vascular disease, age, sex, previous AMI, systolic blood pressure upon admission, and anaemia.

**CONCLUSION:**

As a conclusion, HF seems to be a common occurrence after AMI, in spite of changes in the epidemiological profile of the acute coronary syndrome. An increase in the incidence is registered as well, parallel to a decrease in the mortality following AMI. Attention must be shown for highly risked subpopulations, aged persons, patients with the previous coronary disease, and concomitant conditions.

## Introduction

Heart failure (HF) represents a syndrome due to structural or functional heart disorders, hampering the ability of this organ to sufficiently pump the blood. HF will be accompanied with a shorter life expectancy, as well as other important symptoms such as dyspnea, fluid retention, fatigue and malaise, that will severely worsen the quality of life [1]. Coronary disease is recognised as the main cause of mortality, approximating 20% of all deaths due to cardiovascular conditions in Europe [2]. An improvement in the treatment of AMI, especially following reperfusion therapies, and the increased mean longevity have contributed to an increase of survivals following AMI; the same is valid for patients with HF generally [3-7]. Almost half of the newly diagnosed patients with HF ageing less than 75 years are due to coronary artery disease; and 60% of the patients with HF have a positive history for AMI [8, 9].

HF is a common complication in patients suffering AMI, causing higher costs of treatment and a poor prognosis. Systolic dysfunction of the left ventricle is the main cause of HF following an AMI. The reasons for HF development in the majority of patients following an AMI include the myocardial necrosis and the structural remodelling of the ventricle that pursues. The process of remodelling produces a rapid appearance in the immediate period post-AMI, with the process slowing down thereafter. Studies suggest a bimodal form of HF appearance; the first peak coincides with the hospitalisation, and the second peak the fourth day after admission [10].

HF symptoms are variable, and hardly might one foresee those that lead toward a “chronic” HF. Different factors such as recurrent ischemia, the extent of myocardial necrosis, the so-called “stunned myocardium”, ventricle remodelling, mechanical complications and “hibernating myocardium”, will all play a role in the emergence of systolic ventricular dysfunction post AMI, be it in the setting of a HF or lacking the clinical picture of a HF. In a US study of 2002, it resulted that 20% of AMI patients showed the clinical picture of HF, with 9% having a later appearance [11]. A French study suggested that 38% of AMI patients had the signs of HF within the first five days after the infarction [12]. Several sources suggest a higher HF risk in aged people, patients positive for cardiac diseases and diabetes mellitus [13]. Predictors of an early developing of HF include aged patients, diabetes, previous cardiac symptoms; whereas predictors of a later developing HF include hypertensive disease, male sex, tachycardia and higher values of cardiac enzymes. It seems that HF is more frequent in anterior AMI when compared to other regions of the heart.

There is not much information with regard to the profile of the coronary disease, risk factors, treatment methods and their influence on the prognosis of an AMI [14]. In Albania, a transitional south-eastern European country, only a few studies have approached the issue. A previously published paper aimed towards the evaluation of the incidence, and of the predictors of HF, in a sample population of AMI patients [15].

The present study aimed to assess the incidence of HF developing in patients with AMI admitted and treated in the Coronary Intensive Care Unit of the “Mother Theresa” University Hospital Centre of Tirana, Albania.

## Materials and Methods

We studied the demographics and clinical characteristics of the AMI patients during their admission, as well as the predictors of HF. The present is a prospective study, including the demographic and clinical data of 587 consecutive patients suffering from AMI, from January 2013 till May 2015. Framingham criteria were applied for diagnosing patients with HF upon admission, and a Killip class ≥ 2 was the diagnostic criterion of HF during the period of hospitalisation, I a three-week follow-up period. Valvular, non-ischemic cardiopathy was excluded from the study group.

The data collection included age, gender, educational status, body height and weight, blood pressure, type of myocardial infarction (with ST elevation or in absence of the latter; STEMI vs. NSTEMI [ST Segment Elevation Myocardial Infarction]), localisation, HF complications and the medical treatment applied. Glycemia and lipid profile were performed during fasting. A thorough patient history regarding other risk factors for coronary disease (smoking, hypertension, diabetes, and dyslipidemia) and previous positive history for AMI was collected as well.

The study protocol was approved by the National Committee of Medical Ethics (No. of the protocol 092).

The statistical analysis of discrete (qualitative) variables was performed through presenting the variables in percentage; continuous variables were presented as a mean value ± standard deviation. Groups were compared to each other with a χ^2^ test for discrete variables, and t-test for continuous variables. A logistic regression model was used to determine independent predictors of the HF developing in AMI patients.

## Results

HF was identified in 156 patients (26.6%) of the entire sample group of AMI patients. Epidemiological and clinical characteristics of the group are summarised in Table 1.

**Table 1:** Clinical and epidemiological characteristics of the patients recruited in the present study

Variable	HF present	HF absent	P value
*Socio-demographic profile*			
Number of patients	156	431	
Age, years	69.4 ± 11.2	61.7 ± 11.6	0.001
Sex, male, n (%)	97 (62.3)	355 (82.4)	< 0.0001
BMI, kg/m^2^	26.4±3.7	26.7±2.8	0.381
*Clinical findings on admission*			
Heart rate on admission, bpm	80.16 ± 19.35	70.64 ± 11.13	< 0.001
Systolic BP on admission, mmHg	119.6 ± 30.5	132.9 ± 24.2	0.001
Blood glucose on admission, mg/dl	168.5 ± 83.2	143 ± 77.4	0.007
*AMI location*			
Anterior, n (%)	112 (71.8)	190 (44.1)	<0.0001
Inferior, n (%)	38 (24.4)	192 (44.5)	<0.0001
Presence of STEMI, n (%)	114 (73)	338 (78.4)	0.141
LVEF (%)	36.9 ± 9.1	57.2 ± 5.7	<0 .0001
Previous MI, n (%)	34 (21.8)	29 (6.7)	<0 .0001
Previous HTN treatment, n (%)	55 (35.3)	278 (64.5%)	0.0031
Previous MI, n (%)	34 (21.8)	29 (6.7)	<0 .0001
Previous PCI or CABG, n (%)	16 (10.3)	15 (3.5%)	0.012
*Coronary risk factors*			
Smoking, n (%)	74 (47.4)	196 (45.5%)	0.054
Diabetes, n (%)	52 (33.4)	142 (32.9)	0.114
Dyslipidemia, n (%)	75 (48.1)	207 (48)	0.987
Hypertension, n (%)	125 (80.1)	343 (79.6)	0.923
*Co-morbidity*			
Chronic renal disease, n (%)	19 (12.2)	17 (4)	0.005
Anemia, n (%)	29 (18.6)	34 (7.9)	0.003
Peripheral vascular disease, n (%)	18 (11.5)	15 (3.5)	0.005
AF occurrence, n (%)	13 (8.4)	8 (1.8)	0.003
*In-hospital medication*			
Beta blockers, n (%)	56 (35.9)	292 (67.7)	<0.0001
ACEI or ARB, n (%)	113 (72.4)	315 (73.1)	0.916
Digoxin, n (%)	7 (4.5)	4 (0.9)	0.036
Statins, n (%)	134 (85.9)	380 (88.2)	0.876
Diuretics, n (%)	127 (81.4)	31 (7.2)	<0.0001

Plus – minus values are means ±SD. HF – heart failure; BMI – body mass index; BP – blood pressure; MI – myocardial infarction; HTN – systemic arterial hypertension; PCI – percutaneous coronary intervention; CABG – coronary artery bypass surgery; LVEF – left ventricular ejection fraction; AMI – acute myocardial infarction; STEMI – ST-segment elevation myocardial infarction; AF – atrial fibrillation; ACEI – angiotensin-converting enzyme inhibitor; ARB – angiotensin receptor blocker.

The subgroup of patients with HF showed significant differences from other patients when compared with regard to age, gender (male), heart rate on admission, systolic BP on admission, previous MI, glycemia on admission, previous antihypertensive treatment, previous coronary revascularization procedures, peripheral vascular disease, chronic renal disease, ejection fraction, as well as anemia and for the presence of atrial fibrillation. No significant differences were met with regard to the presence of hypertension (p = 0.923), diabetes (p = 0.114), dyslipidemia (p = 0.987), presence of STEMI (p = 0.141), smoking habits (p = 0.054).

**Figure 1:**
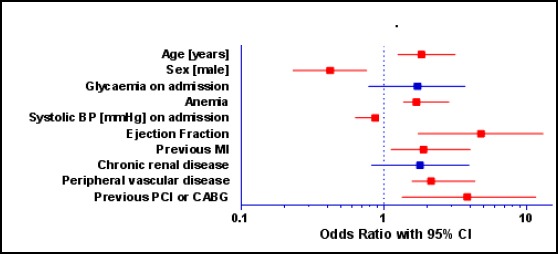
Multivariate analysis of predictors of heart failure in acute myocardial infarction patients

Significant differences in between the two subgroups (with / without HF) were seen with regard to the usage of diuretics, digoxin and beta-blockers. Table 2 represents the multivariate analysis for the predictors of the acute HF in AIM patients. Independent predictors for the HF developing in a logistic regression model were: ejection fraction, previous revascularization, peripheral vascular disease, age, gender, previous myocardial infarction, systolic BP on admission, and anaemia. The data are graphically represented in Figure 1 as well, focusing on the statistical significance of every independent predictor separately.

**Table 2:** Multivariate analysis of the predictors of heart failure, in a group of patients with acute myocardial infarction

Variable	OR (95% CI)	P value
Age [years]	1.83 (1.25 – 3.16)	0.002
Sex [male]	0.42 (0.23 – 0.76)	0.047
Glycemia on admission	1.72 (0.78 – 3.71)	0.211
Systolic BP [mmHg] on admission	0.87 (0.63 – 0.91)	0.032
Ejection Fraction	4.78 (1.73 – 13.03)	0.001
Previous MI	1.89 (1.12 – 4.03)	0.013
Chronic renal disease	1.79 (0.82 – 3.94)	0.0384
Peripheral vascular disease	2.14 (1.57 – 4.36)	0.027
Previous PCI or CABG	3.84 (1.34 – 11.62)	0.0016
Anemia	1.69 (1.37 – 2.85)	0.046

CI – confidence interval; BP – blood pressure; MI – myocardial infarction; OR – odds ratio; PCI – percutaneous coronary intervention; CABG – coronary artery bypass surgery.

## Discussion

HF incidence in our study resulted in 26.6%, whereas other large clinical studies have suggested somehow higher values up to 29.4% (GUSTO I, GUSTO IIb, GUSTO III and ASENT II) [16]. Registry values, including the VALIANT register, suggest figures from 20.4% up to 24.5% [11, 17]. Another large epidemiological study including the period 1970-1999, found that 24% of the AIM cases presented HF [18]. There are a few studies in Albania regarding the factors that might lead to HF developing in the acute phase of AMI [15, 19]. In our present paper, as well as in the Framingham analysis, HF patients were older, they showed a male preponderance, a higher heart rate on admission, higher systolic BP values on admission, positive history for previous MI as well as higher glycemic values on admission, together with more antihypertensive drug usage, an increased presence of previous attempts to coronary revascularization, peripheral vascular disease, chronic renal disease and anemia, a lower ejection fraction and an increased occurrence of atrial fibrillation.

With regard to risk factors, the HF subgroup of patients differed only for the smoking habits. As of the in-patient or intra-hospital treatment, our HF subgroup differed significantly with the subgroup without HF, vis-à-vis digoxin, diuretics and beta-blockers administration. Predictors for an early development of HF were older patients, diabetics, and people suffering from previous cardiac symptoms; whereas predictors for a late-onset HF were hypertensive disease, male gender, tachycardia and higher value of cardiac enzymes. HF was more frequent following anterior AMI when compared with other anatomic cardiac areas.

In our study, independent predictors of the HF developing in a logistic regression model were: ejection fraction value, previous revascularization procedures, peripheral vascular disease, age, gender, previous MI, systolic BP on admission and anaemia. The simultaneous presence of different risk factors and the clinical severity of AMI, together with a suboptimal medical care, will strongly influence the prognosis of the coronary disease. In Albania, there is as well a need for rehabilitation programs, let alone the necessity of a standardisation of interventions aiming at secondary prevention. Obviously, other studies are needed to clarify the importance of factors influencing the clinical profile of Albanian patients suffering an AMI, in order to optimise the quality of care within a coronary intensive care unit.

In conclusion, in spite of a falling trend of mortality linked to myocardial infarction, HF still remains an important complication of the everyday cardiologic practice. In order to better organise prevention and optimal treatment for the HF, an increased attention is needed towards highly risked populations, especially aged persons, those with the previous coronary disease, and other concomitant medical conditions. As a conclusion, we might say that HF is a common occurrence following AMI, notwithstanding changes in the epidemiology of the acute coronary syndrome. In fact, an increased incidence of HF is being reported, parallel to a decrease in the mortality from AMI. Further observations are needed to demonstrate the impact of newly marketed drugs in the HF frequency after an AMI episode so that all requirements of an evidence-based medicine might be accomplished.

**Abbreviations**
AMI –: acute myocardial infarction
MI –: myocardial infarction
BP –: blood pressure
HF –: heart failure
EF –: ejection fraction
STEMI –: ST-Segment Elevation Myocardial Infarction
AF –: atrial fibrillation
